# Development of a Rapid UPLC Method for Analysis of Carotenoids in Goji Berry Extract and Evaluation of Their Transformation Affected by Saponification

**DOI:** 10.3390/molecules29235684

**Published:** 2024-11-30

**Authors:** Bo-Yang Hsu, Chia-Hui Lin, Tsai-Hua Kao

**Affiliations:** 1 Department of Food Science, National Ilan University, Yilan County 260, Taiwan; byhsu@niu.edu.tw; 2 Department of Food Science, Fu Jen University, New Taipei City 242, Taiwan; lindy52099@gmail.com

**Keywords:** goji berry, saponification, carotenoids, zeaxanthin, UPLC-DAD

## Abstract

Goji berry (*Lycium barbarum* L.), also known as wolfberry, is a traditional Chinese medicinal herb widely utilized as a functional food ingredient throughout East Asia. In this study, we developed a rapid high performance liquid chromatography-diode array detection (HPLC-DAD) method for the simultaneous separation of carotenoids in goji berries. This method successfully separates 17 carotenoids and their esters within 21 min using a Sunrise C30 column, with detection at 450 nm, a flow rate of 1.3 mL/min, and a column temperature of 25 °C. Method validation showed intra-day precision ranging from 0.97% to 6.21% and inter-day precision from 0.99% to 7.01%, demonstrating this method effectively minimizes analysis time while providing high separation efficiency and sensitivity. Goji berries extracted with a mixture of n-hexane/ethanol/acetone (1:1:1, *v*/*v*/*v*) and then saponified with a 40% potassium hydroxide methanol solution can completely convert carotenoid esters into free monomer forms. The highest carotenoid content in goji berry was all-*trans*-zeaxanthin (1721.94 ± 81.01 μg/g), followed by 9- or 9′-*cis*-zeaxanthin (79.53 ± 3.92 μg/g), 15- or 15′-*cis*-zeaxanthin (43.71 ± 2.17 μg/g), 9- or 9′-*cis*-zeaxanthin (36.51 ± 1.81 μg/g), all-*trans*-β-cryptoxanthin (25.76 ± 1.55 μg/g), all-*trans*-β-carotene (5.71 ± 0.83 μg/g), and 13- or 13′-*cis*-β-carotene (0.86 ± 0.13 μg/g).

## 1. Introduction

Goji berry (*Lycium barbarum* L.), also known as wolfberry, is the mature fruit of a deciduous shrub within the Solanaceae family. Traditionally, it has been utilized in herbal medicine for its potential benefits in blood nourishment, and in the management of diabetes, tuberculosis, dizziness, cough, and vision health [1,2]. The nutrient profile of goji berries includes 46% carbohydrates, 16% dietary fiber, 13% protein, and 1.5% fat [3]. Additionally, goji berries contain trace levels of essential vitamins and minerals, including vitamins B1 and B2, niacin, magnesium, copper, manganese, and selenium [4,5]. Extensive research has demonstrated various biological activities of Goji berries, such as hypoglycemic effects [6], lipid oxidation inhibition [7], immunomodulatory properties [8], anticancer effects [2], neuroprotective functions [9], and the prevention of retinopathy [10]. As a result, goji berry is widely utilized as a functional food ingredient. The primary bioactive compounds in goji berry include polysaccharides, phenolic compounds, and carotenoids [11,12,13].

Carotenoids are a group of fat-soluble natural pigments consisting of multiple polyisoprene units, responsible for the red, yellow, and orange hues in a variety of fruits and vegetables. Carotenoids are categorized into two primary classes based on structural characteristics: carotenes, which are hydrocarbons (e.g., α-carotene, β-carotene, and lycopene), and xanthophylls, which are oxygenated derivatives (e.g., lutein and zeaxanthin). The hydroxyl groups in xanthophylls increase their polarity, distinguishing them from carotenes, which have relatively lower polarity [14]. Due to their conjugated double bonds, carotenoids can exist in both *cis* and *trans* configurations. In nature, carotenoids predominantly occur in the all-*trans* forms; however, *trans* isomers may convert to *cis* isomers and even degradation on exposure to light, oxygen, heat, and acid [15,16]. For example, studies indicate that all-*trans* zeaxanthin can partially convert into 9-*cis*, 13-*cis*, and 15-*cis* isomers when stored at 75 °C [17]. Additionally, Updike & Schwartz (2003) report that thermal processing of vegetables such as corn, kale, green beans, spinach, and broccoli can lead to up to a 17% formation of *cis*-zeaxanthin [18].

High-performance liquid chromatography (HPLC) is the most widely used method for carotenoid analysis, with reversed-phase chromatography proving particularly effective for carotenoid separation [19]. In column selection, C18 columns are known for their high separation efficiency, especially for carotenoids with structurally similar molecules [19,20]. However, Amorim-Carrilho et al. (2014) note that C30 columns provide superior resolution for separating non-polar carotenoids, such as β-carotene and lycopene, as well as their isomers [21]. Both C18 and C30 columns offer distinct advantages and limitations, making column selection dependent on the specific matrix and characteristics of the carotenoids under study [21]. In terms of the mobile phase, acetonitrile and methanol are the primary solvents employed in carotenoid analysis. Acetonitrile is often preferred for its low viscosity, low UV absorbance, and reduced column pressure, while methanol is valued for its lower toxicity and cost-effectiveness. The addition of low-polarity solvents, such as dichloromethane, ethyl acetate, or *n*-hexane, to the mobile phase can further enhance separation efficiency [21]. Hu et al. (2022) developed an LC-Q-TOF-MS/MS method for carotenoid analysis in goji berries, achieving the successful separation of 13 carotenoids and their isomers within 49 min [22]. Patsilinakos et al. (2018) established an HPLC-DAD method specifically for analyzing zeaxanthin dipalmitate in goji berries with a total analysis time of 35 min [23]. Similarly, Karioti et al. (2014) developed an HPLC-DAD method for the simultaneous analysis of zeaxanthin dipalmitate and β-carotene in goji berries, achieving separation within 30 min [24]. Considering the limited research on carotenoid analysis in goji berries, this study aims to develop a more rapid HPLC-DAD method for simultaneous separation of carotenoids, providing both high resolution and a reduced analysis time.

## 2. Results and Discussion

### 2.1. Evaluation of Different C30 Columns and Gradient Elution System

**Figure 1:**
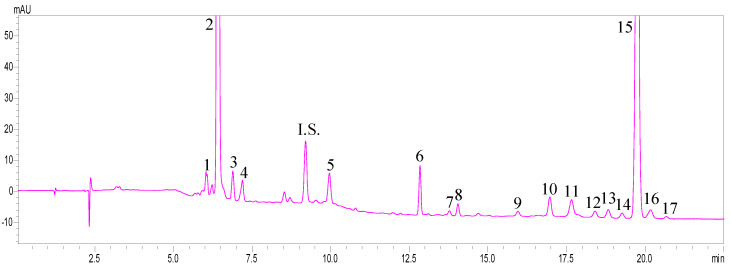
HPLC−DAD chromatogram of carotenoids from goji berry extracts (simultaneous extraction and saponification for 16 h) analyzed by Sunrise C30 (250 × 4.6 mm I.D., particle size 3 μm) column. The mobile phase is A. methanol/acetonitrile/water (84:14:2, *v*/*v*/*v*), B. DCM with flow rate of 1.3 mL/min was used for separation. The detection wavelength is 450 nm. Peak identification: 1. 9-or 9′-*cis*-zeaxanthin; 2. all-*trans*-zeaxanthin; 3. 15-or 15′-*cis*-zeaxanthin; 4. 9-or 9′-*cis*-zeaxanthin; I.S. all-*trans*-β-apo-8′-carotenal; 5. all-*trans*-β-cryptoxanthin; 6. carotenoid esters; 7. 13-or 13′-*cis*-β-carotene; 8. all-*trans*-β-carotene; 9. carotenoid esters; 10. carotenoid esters; 11. carotenoid esters; 12. carotenoid esters; 13. carotenoid esters; 14. carotenoid esters; 15. carotenoid esters.

Compared to C18 columns, C30 columns have longer carbon chains, increasing the thickness of the stationary phase, which enhances the interaction surface area between larger carotenoid molecules and the stationary phase, resulting in better separation of isomers [15]. In recent years, C30 columns have been widely used for separating various carotenoids in different samples. Our previous study developed a method using a YMC C30 (150 × 4.6 mm I.D., particle size 5 µm) column to analyze the largest number of carotenoid types in various samples, which has been broadly applied to carotenoid analysis [2,3,25]. However, the separation time can take 45–52 min. Thus, in this study, we further explored the effect of smaller particle-size C30 columns. After comparing Ascentis Express 160 Å C30 (150 × 4.6 mm I.D., particle size 2.7 µm) with Sunrise C30 (250 × 4.6 mm I.D., particle size 3 µm), we found that, while the Sunrise C30 column is longer and has slightly longer separation times compared to the Ascentis Express 160 Å C30, it offers a more stable baseline and better resolution of isomers. Hence, the Sunrise C30 column was chosen for further evaluation of the optimal separation gradient. In previous studies with C30 columns, the mobile phases for carotenoid separation included different ratios of methanol, acetonitrile, and water mixtures, or methanol-water solutions combined with dichloromethane for separation [2,25,26]. After comparing different mobile phase compositions and gradients, the optimized mobile phase composition and gradient conditions developed in this study were found to be: (A) methanol/acetonitrile/water (84:14:2, *v*/*v*/*v*) and (B) dichloromethane. The starting ratio was 96% A and 4% B, held for 2 min. At the 3rd min, 32% B; at the 7th min, 35% B; at the 8th min, 45% B; at the 11th min, 55% B; at the 16th min, 58% B; at the 17th min, 60% B; at the 20th min, 62% B; at the 21st min, 100% B, held for 3 min. At the 25th min, it returned to 4% B. The flow rate was 1.3 mL/min, the column temperature was 25 °C, and detection was performed at 450 nm. This method could separate 17 carotenoids and their esters from goji berries within 21 min, as shown in Figure 1. We calculated the retention time (tR), retention factor (k), separation factor (α), and peak purity. The k values ranged from 1.57 to 7.65, and the α values were all greater than 1, indicating adequate separation efficiency and separation time for this method of analysis.

Compared with other studies, the current methods for separating carotenoids using C30 columns, including YMC C30 (150 × 4.6 mm I.D., particle size 5 µm), have been able to separate 18 carotenoids from goji berries in 52 min [2,25], and 9 carotenoids in 45 min. Liu et al. (2021) report that 13 carotenoids from Taiwan pomelo leaf extracts were separated in 28 min [27]. The Sunrise C30 column used in this study has smaller packing particles, combined with an increased flow rate, effectively shortening the analysis time while achieving the same carotenoids separation results.

### 2.2. Identification of Carotenoids in Goji Berry

Carotenoid identification in goji berries was achieved by comparing the retention time, absorption spectra, and maximum absorption wavelength of standards with unknown components. Additionally, the Q-ratio for each peak was calculated and compared with the data in the literature. Most carotenoids exhibit a major absorption band in the visible region, consisting of two to three maximum absorption wavelengths and a shoulder. *Cis*-isomers show absorption in the UV region (330–350 nm), resulting in a *cis*-peak, and they cause a spectral shift known as hypsochromic shift (blue shift). The absorbance of the main absorption peak and *cis*-peak varies depending on the isomer, which can be used to identify carotenoid isomers [28]. The Q-ratio is defined as the height ratio of the *cis* peak to the main absorption peak. Different isomers have different Q-ratios, which can serve as a basis for identification.

No commercial carotenoid isomer standards are currently available. This study further promotes the isomerization of commercially available all-*trans* carotenoids standard solution via light exposure, the isomers being separated under the conditions developed earlier. Retention times and absorption spectra of each isomer were established for comparison, and Q-ratios were calculated and compared with the values in the literature.

**Figure 2:**
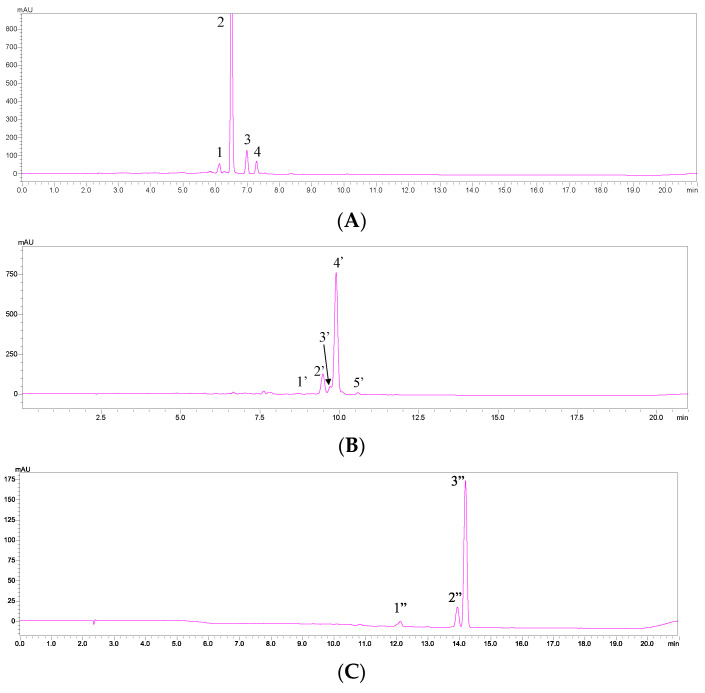
HPLC-DAD chromatogram and absorption spectra of each isomer of (**A**) all-*trans*-zeaxanthin standard, (**B**) all-*trans*-β-cryptoxanthin standard and (**C**) all-*trans*-β-carotene standard after light-induced isomerization. See Table 1 for peak identification.

Figure 2 shows the chromatograms of all-*trans*-zeaxanthin, all-*trans*-β-cryptoxanthin, and all-*trans*-β-carotene after light-induced isomerization, while Table 1 provides retention times, absorption spectra, and Q-ratios for each peak. Table 2 lists the identification data for each peak in the sample. Results from Table 2 indicate that goji berries contain three all-*trans* carotenoids: all-*trans*-zeaxanthin, all-*trans*-β-cryptoxanthin, and all-*trans*-β-carotene, as well as three *cis*-isomers: 9- or 9′-*cis*-zeaxanthin, 15- or 15′-*cis*-zeaxanthin, and 13- or 13′-*cis*-β-carotene. Peaks 6 and 9-17 did not match with any known light-induced isomers or other all-*trans* standards, but their maximum absorption wavelengths and spectra resembled those of carotenoids.

Several studies have indicated that zeaxanthin exists mainly in esterified form in fruits and vegetables [29], and in goji berries, zeaxanthin dipalmitate can account for up to 77.5% of the total carotenoid content in ripe fruits [4]. Other studies have found that the un-saponified carotenoid extract from goji berries contains the highest amounts of zeaxanthin dipalmitate, followed by β-cryptoxanthin monopalmitate and its two isomers, zeaxanthin monopalmitate and its two isomers, along with small amounts of all-*trans*-β-carotene and all-*trans*-zeaxanthin [11]. Thus, it is speculated that peaks 6 and 9–17 are carotenoid esters. However, even with mass spectrometry, the identification of esterified compounds remains challenging due to the lack of standard references. Moreover, free carotenoids are known to be more readily absorbed than their esterified counterparts, which prompted further investigation using a saponification step to remove ester groups, converting all carotenoids to their free form, and evaluating the saponification conditions to achieve the highest free carotenoid content.

**Table 1:** Identification data of all-trans-zeaxanthin standard, all-*trans*-β-cryptoxanthin standard and all-*trans*-β-carotene after light-induced isomerization.

Peak No.	Carotenoids	t_R_ (min)	Absorption Wavelength (nm)		Q-Ratio	
			In-Line	Reported	Found	Reported
1	9- or 9′-*cis*-zeaxanthin	6.088	344 425 449 476	340 42 450 474 ^a^	0.07	0.09 ^a^
2	all-*trans*-zeaxanthin	6.520	428 455 480	-	-	-
3	15- or 15′-*cis*-zeaxanthin	6.997	340 424 449 473	338 424 448 476 ^b^	0.43	0.45 ^b^
4	9- or 9′-*cis*-zeaxanthin	7.299	345 424 449 475	340 424 450 474 ^a^	0.07	0.09 ^c^
1′	9- or 9′-*cis*-β-cryptoxanthin	8.927	430 449 476	346 424 450 476 ^a^	0.15	0.11 ^a^
2′	13- or 13′-*cis*-β-cryptoxanthin	9.492	342 428 450 475	340 422 446 474 ^b^	0.30	0.30 ^b^
3′	13- or 13′-*cis*-β-cryptoxanthin	9.727	342 428 449 475	340 422 446 474 ^b^	0.30	0.30 ^b^
4′	all-*trans*-β-cryptoxanthin	9.910	430 456 481	-	-	-
5′	9- or 9′-*cis*-β-cryptoxanthin	10.596	344 430 452 477	346 424 450 476 ^a^	0.08	0.11 ^a^
1″	9- or 9′-*cis*-β-carotene	12.134	428 454 482	342 426 448 476 ^a^	0.12	0.11 ^a^
2″	13- or 13′-*cis*-β-carotene	13.795	344 428 452 476	342 422 450 474 ^b^	0.46	0.43 ^a^
3″	all-*trans*-β-carotene	14.212	430 458 483	-	-	-

^a^ Based on a reference by Inbaraj et al. (2008) [25]. ^b^ Based on a reference by Hsu et al. (2017) [26]. ^c^ Based on a reference by Hsu and Chen. (2022) [30]. Q-ratio is defined as the *cis* peak (320–350 nm) to the main absorption peak (430–460 nm).

**Table 2:** Retention time, absorption wavelength and Q-ratio of carotenoids in goji berries.

Peak No.	Carotenoids	t_R_ (min)	Absorption Wavelength (nm)			Q-Ratio	
			In-Line	Standard	Reported	Found	Reported
1	9-or 9′-*cis*-zeaxanthin	6.155	346 425 449 476	344 425 449 476	340 424 450 474 ^a^	0.07	0.08 ^b^
2	all-*trans*-zeaxanthin	6.515	428 455 480	428 455 480	-	-	-
3	15-or 15′-*cis*-zeaxanthin	6.977	341 424 449 473	340 424 449 473	338 424 448 476 ^c^	0.43	0.45 ^a^
4	9-or 9′-*cis*-zeaxanthin	7.236	345 424 449 475	345 424 449 475	340 424 450 474 ^a^	0.10	0.12 ^a^
I.S.	all-*trans*-β-apo-8′-carotenal	9.430	-	471	-	-	-
5	all-*trans*-β-cryptoxanthin	10.020	428 456 481	430 456 481	-	-	-
6	carotenoid esters	12.866	345 431 457 483	-	-	-	-
7	13-or 13′-*cis*-β-carotene	13.736	346 428 452 476	344 428 452 476	342 422 450 474 ^c^	0.46	0.43 ^a^
8	all-*trans*-β-carotene	14.058	430 458 484	430 458 483	-	-	-
9	carotenoid esters	16.002	426 447 475	-	-	-	-
10	carotenoid esters	17.027	348 430 457 483	-	-	-	-
11	carotenoid esters	17.730	338 430 452 479	-	-	-	-
12	carotenoid esters	18.471	339 434 457 483	-	-	-	-
13	carotenoid esters	18.885	344 434 457 483	-	-	-	-
14	carotenoid esters	19.329	342 434 455 482	-	-	-	-
15	carotenoid esters	20.037	346 431 457 483	-	-	-	-
16	carotenoid esters	20.228	342 428 452 475	-	-	-	-
17	carotenoid esters	20.727	332 431 456 483	-	-	-	-

^a^ Based on a reference by Inbaraj et al. (2008) [25]. ^b^ Based on a reference by Liu et al. (2021) [27]. ^c^ Based on a reference by Hsu et al. (2017) [2]. I.S. = internal standard. Q-ratio is defined as the *cis* peak (320–350 nm) to the main absorption peak (430–460 nm).

### 2.3. Effect of Saponification on the Transformation of Carotenoids in Goji Berries

Saponification can remove impurities such as chlorophyll and lipids, and is one of the common methods used for the purification of carotenoids [31]. Saponification can either be performed simultaneously during sample extraction (simultaneous saponification) or after extraction (post-extraction saponification). Depending on the conditions for saponification (time, temperature, concentration of potassium hydroxide, etc.), carotenoids may undergo varying degrees of isomerization and degradation, with prolonged saponification leading to degradation [32]. Although saponification increases time and experimental costs, it can remove various impurities, hydrolyze carotenoid esters into free forms of carotenoids such as lutein [25], aid in the qualitative analysis of carotenoids and transform them into forms with higher absorbance. Below is a discussion of the effects of post-extraction hot saponification, simultaneous saponification, and post-extraction room temperature saponification in this study.

#### 2.3.1. Extraction Followed by Heat Saponification

**Figure 3:**
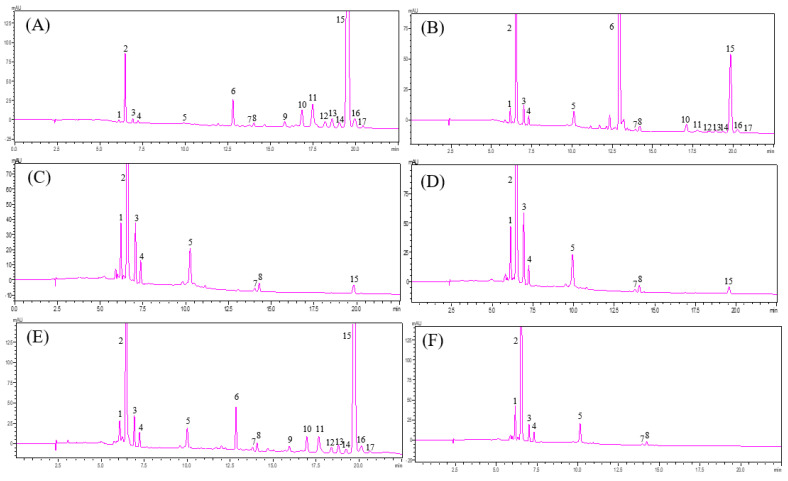
HPLC-DAD chromatograms of carotenoids from goji berry extracts with different degrees of saponification. (**A**) without saponification, (**B**) extraction followed by heat saponification at 56 °C for 20 min, (**C**) extraction followed by heat saponification at 56 °C for 40 min, (**D**) extraction followed by heat saponification at 56 °C for 60 min, (**E**) simultaneous extraction and saponification for 16 h, (**F**) extraction followed by saponification in room temperature for 6 h. See Table 2 for peak identification.

This study replicated the conditions for the saponification of carotenoids in goji berry extracts by Hsu et al. (2017), using hot saponification at 56 °C for 20 min [2]. The results, as shown in Figure 3B, compared to Figure 3A (without saponification), showed an increase in the area of peak 6 and a decrease in the area of peaks 9–17, suggesting that esters were partially hydrolyzed but saponification was still incomplete. Therefore, the heating times were further increased to 40 and 60 min, as shown in Figure 3C,D. Only peak 15 remained, with other peaks disappearing, while peaks 1–5, 7, and 8 significantly increased. Thus, it is inferred that most of the esters had been saponified and converted into free-form carotenoids, including all-*trans* carotenoids and their *cis* isomers. Since the above conditions still did not result in complete saponification, further comparisons of other saponification methods were made.

#### 2.3.2. Simultaneous Saponification

From the results in Figure 3E, it can be seen that after saponification under these conditions, the decrease in the area of peaks 6 and 9–17 was minimal, indicating the least effective saponification. It is speculated that the high content of esterified carotenoids in goji berries caused solid clumping during the long saponification process, preventing sufficient contact between KOH and the sample, thus inhibiting the saponification process.

#### 2.3.3. Extraction Followed by Saponification in Room Temperature

Finally, following the method of Inbaraj et al. (2008) [25], saponification was carried out at 25 °C for 6 h after solvent extraction. As shown in Figure 3F, peaks 6 and 9–17 completely disappeared, while peaks 1–5, 7, and 8 significantly increased compared to the unsaponified sample (Figure 3A), indicating that all the esterified carotenoids had been converted into free-form all-*trans* and *cis* isomers. The chromatogram reduced from 17 peaks (unsaponified) to 7 peaks, all of which could be matched with either standard samples or isomers produced by photoisomerization. Based on the above results, saponification using a 40% KOH-methanol solution at 25 °C for 6 h following extraction can convert all esterified carotenoids in the goji berry extract into free monomers.

### 2.4. Method Validation

Table 3 presents the intra- and inter-day precision for the analysis of various carotenoids and their isomers in saponified goji berry extracts. The intra-day precision ranged from 0.97% to 6.21%, while the inter-day precision ranged from 0.99% to 7.01%, indicating high repeatability of the analytical method. Since the related literature indicates that *cis* isomers do not have commercially available standards, the relative quantification of *cis* isomers was performed using all-*trans* standards. The detection limits (LOD) for all-*trans*-zeaxanthin, all-*trans*-β-cryptoxanthin, and all-*trans*-β-carotene developed in this study were 0.010, 0.017, and 0.016 μg/mL, respectively, with quantification limits (LOQ) of 0.033, 0.055, and 0.053 μg/mL, respectively (Table 3). In previous similar studies, Kao et al. (2011) report detection limits of 0.025, 0.025, and 0.05 μg/mL for all-*trans*-zeaxanthin, all-*trans*-β-cryptoxanthin, and all-*trans*-β-carotene, respectively, with quantification limits of 0.075, 0.075, and 0.15 μg/mL, respectively [33]. Additionally, Hsu and Chen (2022) report detection limits of 0.1, 0.025, and 0.05 μg/mL for the aforementioned all-*trans*-carotenoids, with quantification limits of 0.3, 0.075, and 0.15 μg/mL [30]. These values are higher than those obtained in this study, possibly due to the smaller packing particles used in the column, resulting in better separation, narrower peaks, and higher sensitivity. Since this study adopted the previously developed extraction method, recovery rate evaluation was not performed. However, based on prior evaluations, this method demonstrated high recovery rates (82.8–88.5%) [2].

### 2.5. Quantification of Carotenoids in Goji Berry

Strati et al. (2012) indicate that, due to the similar extinction coefficients, *cis*-isomers of carotenoids can be quantified using the standard curve of their corresponding all-*trans*-carotenoids [34]. Consequently, this study also used the standard curve of all-*trans* carotenoids for the quantification of carotenoids in goji berries. The R² values in Table 3 demonstrate a high linear correlation between concentration and peak area for the standard curves. In this study, the internal standard method was used to quantify carotenoids in a goji berry extract, which was saponified for 6 h after extraction. The peak areas from the analysis were substituted into the standard curves to calculate the content, which was then multiplied by the dilution factor and quantified volume, and divided by the sample weight to obtain the content of seven carotenoids and their *cis* isomers in the extract. The results are shown in Table 4. The highest content was found for all-*trans*-zeaxanthin (1721.94 ± 81.01 μg/g), followed by 9- or 9′-*cis*-zeaxanthin (79.53 ± 3.92 μg/g), 15- or 15′-*cis*-zeaxanthin (43.71 ± 2.17 μg/g), 9- or 9′-*cis*-zeaxanthin (36.51 ± 1.81 μg/g), all-*trans*-β-cryptoxanthin (25.76 ± 1.55 μg/g), all-*trans*-β-carotene (5.71 ± 0.83 μg/g), and 13- or 13′-*cis*-β-carotene (0.86 ± 0.13 μg/g). From these results, it can be concluded that the total amount of zeaxanthin (including its isomers) is the highest in goji berries after saponification, approximately 1882 μg/g, followed by β-cryptoxanthin (approximately 26 μg/g) and β-carotene (approximately 7 μg/g). This result is consistent with the findings of Hsu et al. (2017) and Inbaraj et al. (2008) [2,25].

Hsu et al. (2017), using hexane/ethanol/acetone (1:1:1, *v*/*v*/*v*) for extraction, found that a saponified Goji berry extract contained approximately 1508 μg/g of zeaxanthin, 47.2 μg/g of β-cryptoxanthin, and 16 μg/g of β-carotene [2]. Inbaraj et al. (2008), using hexane/ethanol/acetone/toluene (10:6:7:7, *v*/*v*/*v*/*v*), found that a saponified Goji berry extract contained approximately 1266 μg/g of zeaxanthin, 53 μg/g of β-cryptoxanthin, and 32 μg/g of β-carotene [25]. Both studies reported lower zeaxanthin content than found in this study, but higher β-cryptoxanthin and β-carotene content. The differences are likely due to the variation in goji berry sources, varieties, regions, or seasons, as well as differences in extraction solvents and saponification methods. However, a consistent finding is that zeaxanthin > β-cryptoxanthin > β-carotene in the carotenoid content of goji berry extracts.

## 3. Materials and Methods

### 3.1. Materials

Goji berries were purchased from a traditional Chinese medicine store in New Taipei City, Taiwan and stored at −20 °C in the dark. All-*trans*-zeaxanthin, all-*trans*-α-carotene and all-*trans*-β-apo-8′-carotenal (internal standard) were purchased from ChromaDex (Los Angeles, CA, USA). All-*trans*-β-cryptoxanthin and all-*trans*-lutein were obtained from Extrasynthese (Genay, France). All-*trans*-β-carotene was purchased from Nacalai Tesque (Kyoto, Japan). All-*trans*-lycopene was purchased from Cayman Chemical (Ann Arbor, MI, USA). Methanol, dichloromethane and acetone were purchased from Avantor (Radnor Township, PA, USA). Hexane was obtained from Duksan (Gyeonggido, Republic of Korea). Acetonitrile and sodium sulfate anhydrous were purchased from Honeywell (Charlotte, NC, USA). Ethanol at 95% was purchased from Echochemical Co. (Miaoli, Taiwan). Potassium hydroxide was obtained from Thermo Fisher Scientific (Hampton, NH, USA).

### 3.2. Evaluation of UPLC Columns

Two C30 columns, the Sunrise C30 (250 × 4.6 mm, 3 μm, ChromaNik Technologies, Osaka, Japan) and the Ascentis Express C30 (150 × 4.6 mm, 2.7 μm, Merck KGaA, Darmstadt, Germany), were employed to compare the separation efficiency of carotenoids in Goji berries. The evaluation was based on the method described by Hsu et al. (2012) [26]. A Shimadzu UPLC system (Kyoto, Japan) equipped with a Nexera X2 LC-30AD chromatographic pump, a Nexera X2 SPD-M30A diode array detector (DAD), and a binary solvent system comprising (A) methanol/acetonitrile/water (84:14:4, *v*/*v*/*v*) and (B) dichloromethane was used at a flow rate of 1 mL/min. The solvent gradient conditions were set as follows: starting with 100% A and 0% B, increasing to 10% B at 4 min, 18% B at 12 min, 21% B at 17 min, 30% B at 20 min (maintained for 5 min), 39% B at 28 min, 60% B at 40 min, and reaching 100% B at 45 min. The column temperature was maintained at 25 °C, and detection was conducted at a wavelength of 450 nm.

### 3.3. UPLC-DAD Analysis of Carotenoids

A modified method based on Hsu et al. (2012) was employed for the determination of carotenoids in goji berries [26]. The analysis was performed using a Shimadzu UPLC system (Kyoto, Japan) equipped with a Nexera X2 LC-30AD chromatographic pump and a Nexera X2 SPD-M30A diode array detector (DAD). A Sunrise C30 column (250 × 4.6 mm, 3 μm, ChromaNik Technologies, Osaka, Japan) was utilized with a binary solvent system consisting of (A) methanol/acetonitrile/water (84:14:2, *v*/*v*/*v*) and (B) dichloromethane, at a flow rate of 1.3 mL/min for carotenoid separation. The solvent gradient was programmed as follows: 96% A and 4% B initially, maintained for 2 min, increasing to 32% B at 3 min, 35% B at 7 min, 45% B at 8 min, 55% B at 11 min, 58% B at 16 min, 60% B at 17 min, 62% B at 20 min, and finally 100% B at 21 min, held for 3 min. The column temperature was maintained at 25 °C, and detection was performed at a wavelength of 450 nm.

### 3.4. Identification and Photoisomerization of Carotenoids

Carotenoid identification in goji berries was conducted by comparing the retention times and absorption spectra of unknown peaks with those of reference standards and with data reported in the literature. To identify *cis*-isomers of carotenoids, standards of all-*trans*-zeaxanthin, all-*trans*-β-cryptoxanthin, and all-*trans*-β-carotene were individually dissolved in dichloromethane at a concentration of 500 μg/mL in glass vials. The vials were placed in an incubator at 25 °C and illuminated for 24 h at a light intensity of 2000–3000 Lux to induce photoisomerization. Following illumination, each standard solution was filtered through a 0.22 μm PTFE membrane filter prior to UPLC analysis. The UV spectra of each photoisomerized carotenoid standard peak were then compared with those of unknown peaks on the UPLC chromatogram of the sample.

### 3.5. Effect of Saponification on the Transformation of Carotenoids

#### 3.5.1. Extraction Without Saponification

The carotenoid extraction method was adapted from Inbaraj et al. (2008) with modifications to optimize extraction from goji berries [25,26]. The procedure was performed under dark conditions to minimize carotenoid isomerization and degradation. A mixture of 1 g of goji berry pulp and 20 mL of a hexane/ethanol/acetone solution (1:1:1, *v*/*v*/*v*) was shaken at 2750 rpm for 1 h at room temperature. Afterward, 15 mL of a 10% anhydrous sodium sulfate solution was added and vortexed for 5 min. The mixture was then centrifuged at 4000 rpm for 5 min at 4 °C. The supernatant was collected, and the residue was re-extracted with 15 mL of hexane. This extraction procedure was repeated until the extract appeared colorless. All supernatants were pooled and evaporated to dryness under vacuum, and the residue was dissolved in 1 mL of dichloromethane. The sample solution was filtered through a 0.22 μm PTFE membrane filter and stored in a brown vial at −20 °C.

#### 3.5.2. Simultaneous Extraction and Saponification

A mixture of 1 g of goji berry pulp and 20 mL of a hexane/ethanol/acetone solution (1:1:1, *v*/*v*/*v*) was shaken in a brown vial to prevent light exposure for 1 h at room temperature. Next, 1 mL of 40% methanolic potassium hydroxide was added, and the vial was filled with nitrogen gas to prevent carotenoid isomerization or degradation during saponification, which proceeded in the dark for 12 h. Subsequently, 15 mL of a 10% anhydrous sodium sulfate solution was added and vortexed for 5 min. The mixture was then centrifuged at 4000 rpm for 5 min at 4 °C. The supernatant was collected, and the residue was re-extracted with 15 mL of hexane. This extraction procedure was repeated until the extract appeared colorless. All supernatants were pooled and evaporated to dryness under vacuum, and the residue was dissolved in 1 mL of dichloromethane. The sample solution was filtered through a 0.22 μm PTFE membrane filter and stored in a brown vial at −20 °C.

#### 3.5.3. Extraction Followed by Saponification

A mixture of 1 g of goji berry pulp and 20 mL of a hexane/ethanol/acetone solution (1:1:1, *v*/*v*/*v*) was shaken in a brown vial at 2750 rpm for 1 h at room temperature. Subsequently, 15 mL of a 10% anhydrous sodium sulfate solution was added and vortexed for 5 min. The mixture was then centrifuged at 4000 rpm for 5 min at 4 °C. The supernatant was collected, and the residue was re-extracted with 15 mL of hexane. This extraction was repeated until the extract appeared colorless. All supernatants were pooled and evaporated to dryness under vacuum. The dried residue was dissolved in 20 mL of hexane/ethanol/acetone (1:1:1, *v*/*v*/*v*) and mixed with 1 mL of 40% methanolic potassium hydroxide for saponification. For saponification under elevated temperatures, the mixture was maintained at 56 °C for 20, 40, and 60 min in the dark under nitrogen. For saponification at room temperature, the mixture was shaken for 2 h and then kept in the dark under nitrogen for an additional 4 h, resulting in a total saponification time of 6 h. Following saponification, 15 mL of 10% anhydrous sodium sulfate solution was added and vortexed for 5 min. The mixture was centrifuged at 4000 rpm for 5 min at 4 °C. The supernatant was collected, and the residue was re-extracted with 15 mL of hexane, repeating this extraction until the extract was colorless. All supernatants were pooled and evaporated to dryness under vacuum. The final residue was dissolved in 1 mL of dichloromethane, filtered through a 0.22 μm PTFE membrane filter, and stored in a brown vial at −20 °C.

### 3.6. Method Validation

#### 3.6.1. Reproducibility

The method for evaluating intra-day and inter-day variability was adapted from Hsu et al. (2017) [2]. Intra-day variability was assessed by analyzing the carotenoid content in a Goji berry extract sample containing 10 μg/mL of internal standard. This analysis was performed in the morning, afternoon, and evening on the same day, for a total of 9 iterations. Inter-day variability was evaluated by analyzing the carotenoids content in the goji berry extract sample (also with 10 μg/mL of internal standard) in 3 iterations each day over three consecutive days. Intra-day and inter-day variabilities were expressed as the relative standard deviation (RSD, %) for each carotenoid concentration within the same day and across days, respectively.

#### 3.6.2. Detection and Quantitation Limits

To determine the limit of detection (LOD) and limit of quantitation (LOQ), the method described by Inbaraj et al. (2008) was employed [25]. Two concentrations (0.1 and 0.25 μg/mL) of each carotenoid standard were analyzed in triplicate. The LOD and LOQ for each carotenoid were calculated based on signal-to-noise ratios (S/N) of 3 and 10, respectively.

### 3.7. Quantification of Carotenoids in Goji Berries Extract

To prepare stock solutions (1000 μg/mL), three carotenoid standards (all-*trans*-zeaxanthin, all-*trans*-β-cryptoxanthin, and all-*trans*-β-carotene) along with the internal standard (all-*trans*-β-apo-8′-carotenal) were dissolved in dichloromethane. These stock solutions were further diluted with dichloromethane to prepare standard calibration curves. Six concentrations were prepared for all-*trans*-β-cryptoxanthin and all-*trans*-β-carotene (0.5, 1, 5, 10, 20, and 30 μg/mL) and for all-*trans*-zeaxanthin (0.5, 1, 10, 25, 50, and 75 μg/mL). Each diluted standard solution was mixed with the internal standard (all-*trans*-β-apo-8′-carotenal) to achieve a final internal standard concentration of 10 μg/mL. Standard calibration curves were generated by plotting the ratio of the peak area of each carotenoid standard (As) to the peak area of the internal standard (Ai) against the ratio of the concentration of the standard (Cs) to the concentration of the internal standard (Ci). Linear regression and coefficients of determination (R²) for each calibration curve were obtained automatically. Carotenoid content in Goji berry samples were determined by calculating each compound’s concentration using its respective linear regression.

### 3.8. Statistical Analysis

Statistical analyses were conducted using the TIBCO Statistica 14.0.0.15 software. Analysis of variance (ANOVA) and Duncan’s multiple range test were performed to determine significant differences, with a significance threshold set at *p* < 0.05.

## 4. Conclusions

This study evaluated the analysis of carotenoids using a C30 column with smaller packing particles. The results showed that the Sunrise C30 (250 × 4.6 mm I.D., 3 μm) column, with a mobile phase of (A) methanol/acetonitrile/water (84:14:2, *v*/*v*/*v*) and (B) 100% methyl tert-butyl ether, separated all-*trans*-carotenoids, including their *cis*-isomers, from goji berries with higher resolution, improving sensitivity. Saponification can transform carotenoid esters into free forms. The highest content of carotenoids after saponification was zeaxanthin, followed by β-cryptoxanthin and β-carotene.

## Figures and Tables

**Table 3:** Precision (%) data of carotenoids in goji berry extracts by HPLC-DAD analysis and limit of detection (LOD) and limit of quantitation (LOQ) of carotenoid standards.

Peak No.	Carotenoids	Inter-Day Variability ^a^		Intra-Day Variability ^a^		LOD ^c^ (μg/mL)	LOQ ^d^ (μg/mL)	Test Range (μg/mL)	R^2^
		Content (μg/mL)	RSD (%) ^b^	Content (μg/mL)	RSD (%) ^b^				
1	9- or 9′-*cis*-zeaxanthin	78.21 ± 1.97	2.52	77.06 ± 1.18	1.53	-	-	-	
2	all-*trans*-zeaxanthin	1780.34 ± 32.8	1.84	1724.75 ± 15.66	0.91	0.010	0.033	0.5–75	0.999
3	15- or 15′-*cis*-zeaxanthin	44.89 ± 1.42	3.17	46.75 ± 1.21	2.58	-	-	-	
4	9- or 9′-*cis*-zeaxanthin	37.59 ± 0.37	0.99	37.69 ± 0.36	0.97	-	-	-	
5	all-*trans*-β-cryptoxanthin	25.82 ± 0.41	1.59	25.20 ± 0.38	1.53	0.017	0.055	0.5–30	0.997
7	13- or 13′-*cis*-β-carotene	0.90 ± 0.06	7.01	0.87 ± 0.05	6.21	-	-	-	
8	all-*trans*-β-carotene	5.73 ± 0.15	2.66	5.44 ± 0.13	2.44	0.016	0.053	0.5–30	0.998

^a^ Calculated by nine replicates. ^b^ RSD (%) = (SD/mean) × 100%. ^c^ LOD based on S/N = 3. ^d^ LOQ based on S/N = 10. Data are presented as mean ± standard deviation.

**Table 4:** Content of carotenoids in goji berries.

Peak No.	Carotenoids	Content (μg/g)
1	9- or 9′-*cis*-zeaxanthin	79.53 ± 3.92
2	all-*trans*-zeaxanthin	1721.94 ± 81.01
3	15- or 15′-*cis*-zeaxanthin	43.71 ± 2.17
4	9- or 9′-*cis*-zeaxanthin	36.51 ± 1.81
5	all-*trans*-β-cryptoxanthin	25.76 ± 1.55
7	13- or 13′-*cis*-β-carotene	0.86 ± 0.13
8	all-*trans*-β-carotene	5.71 ± 0.83

Data are presented as mean ± standard deviation.

## Data Availability

The data presented in this study are available in this article.
